# The development of cancers research based on mitochondrial heat shock protein 90

**DOI:** 10.3389/fonc.2023.1296456

**Published:** 2023-11-30

**Authors:** Yuchu Xiang, Xudong Liu, Qi Sun, Kuo Liao, Xiaohan Liu, Zihui Zhao, Lishuang Feng, Yan Liu, Bo Wang

**Affiliations:** ^1^ West China Hospital of Sichuan University, Sichuan University, Chengdu, China; ^2^ Institute of Medical Microbiology and Hygiene, Faculty of Medicine, University of Freiburg, Freiburg, Germany; ^3^ Department of Pharmacology and Pharmacy, Li Ka Shing Faculty of Medicine, Centre for Safe Medication Practice and Research, The University of Hong Kong, Pok Fu Lam, Hong Kong SAR, China; ^4^ School of Biology and Biological Engineering, South China University of Technology, Guangzhou, China; ^5^ Multiscale Research Institute of Complex Systems, Fudan University, Shanghai, China; ^6^ Shanghai Jiao Tong University School of Medicine, Shanghai, China; ^7^ School of Public Health, Li Ka Shing Faculty of Medicine, The University of Hong Kong, Hong Kong, Hong Kong SAR, China; ^8^ Department of Organ Transplantation, Guizhou Provincial People’s Hospital, Guiyang, Guizhou, China; ^9^ Department of Urology, Guizhou Provincial People’s Hospital, Guiyang, Guizhou, China

**Keywords:** mtHsp90, TRAP1, molecular chaperone, mitochondria, gamitrinib

## Abstract

Mitochondrial heat shock protein 90 (mtHsp90), including Tumor necrosis factor receptor-associated protein 1 (TRAP1) and Hsp90 translocated from cytoplasm, modulating cellular metabolism and signaling pathways by altering the conformation, activity, and stability of numerous client proteins, and is highly expressed in tumors. mtHsp90 inhibition results in the destabilization and eventual degradation of its client proteins, leading to interference with various tumor-related pathways and efficient control of cancer cell development. Among these compounds, gamitrinib, a specific mtHsp90 inhibitor, has demonstrated its safety and efficacy in several preclinical investigations and is currently undergoing evaluation in clinical trials. This review aims to provide a comprehensive overview of the present knowledge pertaining to mtHsp90, encompassing its structure and function. Moreover, our main emphasis is on the development of mtHsp90 inhibitors for various cancer therapies, to present a thorough overview of the recent pre-clinical and clinical advancements in this field.

## 1 Introduction

Heat shock protein 90 (Hsp90) is a highly conserved family of molecular chaperones that serves a pivotal function in the processes of protein folding, stabilization, and degradation. With several hundred protein clients, Hsp90 is involved in various cellular processes, such as signal transduction, protein trafficking, and DNA repair (1). It functions as an ATP-dependent molecular chaperone, and it’s the crucial role of facilitating the appropriate folding, conformational alteration, activation, and regulation of client proteins. During its functional process, it combines and hydrolyzes ATP, while also involving client proteins and co-chaperones, which are proteins responsible for regulating the activity and function of Hsp90 (2). Hsp90 exhibits distinct localization patterns inside diverse cellular compartments, each associated with specific functional tasks.

In mammalian cells, the Hsp90 family consists of four highly conserved isoforms: the endoplasmic reticulum-localised Grp94, the cytoplasmic Hsp90α and Hsp90β, and TRAP1, which was found in mitochondria (3). These different members exhibit a common molecular chaperone activity pattern, but they selectively bind to diverse client proteins, which is influenced in part by their cellular localization (4). TRAP1 is a Hsp90 homolog and the predominant chaperone in the mitochondria of cancer cells (5). Particularly, Hsp90 compartmentalized in mitochondria is essential regulator of bioenergetics in tumor cells but not normal cells (6). Though many evidences indicated mtHsp90 could be a new potential antitumor target, there is not a review updating its antitumor mechanism and the newest advancements in clinical therapy development.

Due to the increasing number of members of various human heat shock protein (HSP) families, inconsistencies in their nomenclature often result in ambiguity and confusion. In 2009, Kampinga et al. proposed Guidelines for the nomenclature of the human heat shock proteins (7). Among them, the Hsp90 family was renamed as the HSPC family, with the original Hsp90 referred to as HSPC1, Hsp90α and Hsp90β as HSPC2 and HSPC3, respectively, and GRP94 renamed as HSPC4 whereas TRAP1/Hsp75 was renamed as HSPC5. We have summarized the agreement of different scholars on the name of Hsp90 through extensive literature review, and hereby we would like to define the concept of Hsp90 mentioned in the article.

Broadly, Hsp90 can refer to all proteins in the Hsp90 family, whereas narrowly, Hsp90 refers to the classical Hsp90α and Hsp90β. Hoter et al. (8) in 2018 summarized, based on available studies, that Hsp90 has been customarily used for Hsp90α and Hsp90β (9, 10). We have reached the same conclusion through an extensive review of the literature from earlier years. Throughout the discovery history of the Hsp90 family, sequence information for Hsp90α and Hsp90β was first revealed in 1984 (11), whereas grp94 was first sequenced in 1986 (12), and TRAP1 in 1995 (13). Since Hsp90α and Hsp90β in the cytoplasm were first identified, they have been referred to as Hsp90 by researchers in the subsequent literature, whereas Hsp90 homologous proteins in the endoplasmic reticulum and mitochondria are denoted by grp94 and TRAP1, respectively. Typically, without specific indication, Hsp90 is referred to as narrowly defined Hsp90, i.e., Hsp90α and Hsp90β in the cytoplasm.

In 2007, Kang et al. (14) showed that Hsp90 and its related molecule TRAP1 were abundantly present in the mitochondria of tumor cells, and that Hsp90 was not present in normal mitochondria but was prevalent in the cytoplasm of both normal and tumor cells. However, the exact mechanism of how Hsp90 is imported into mitochondria remains to be fully elucidated. In addition, Boucherat et al, in their study of mitochondrial Hsp90 accumulation and vascular remodeling in pulmonary arterial hypertension, also mentioned that Hsp90 is present in tumor cell mitochondria in addition to being highly expressed in the cytoplasm. Boucherat referred to Hsp90 in mitochondria uniformly as mtHsp90 in his paper [9]. Taken together, we refer to Hsp90 in mitochondria as mtHsp90 for short in our paper, including TRAP1 as well as Hsp90 imported from the cytoplasm.

In this review, our primary focus is on mtHsp90, given its unique subcellular location and potential applications in the development of cancer treatments. In the context of cancer cells, mtHsp90, specifically TRAP1, has the potential to function as either a proto-oncogene or an oncogene (15), though it is commonly observed to be overexpressed in various cancer types, including prostate, breast, lung, and leukemia (16). In addition, we will introduce the basic molecular structure and interaction mechanisms of mtHsp90. Subsequently, we will mainly focus on the application of mtHsp90 inhibitors in cancer treatment.

## 2 The structure and function mechanisms of mtHsp90

### 2.1 The structure of mtHsp90

**Figure 1:**
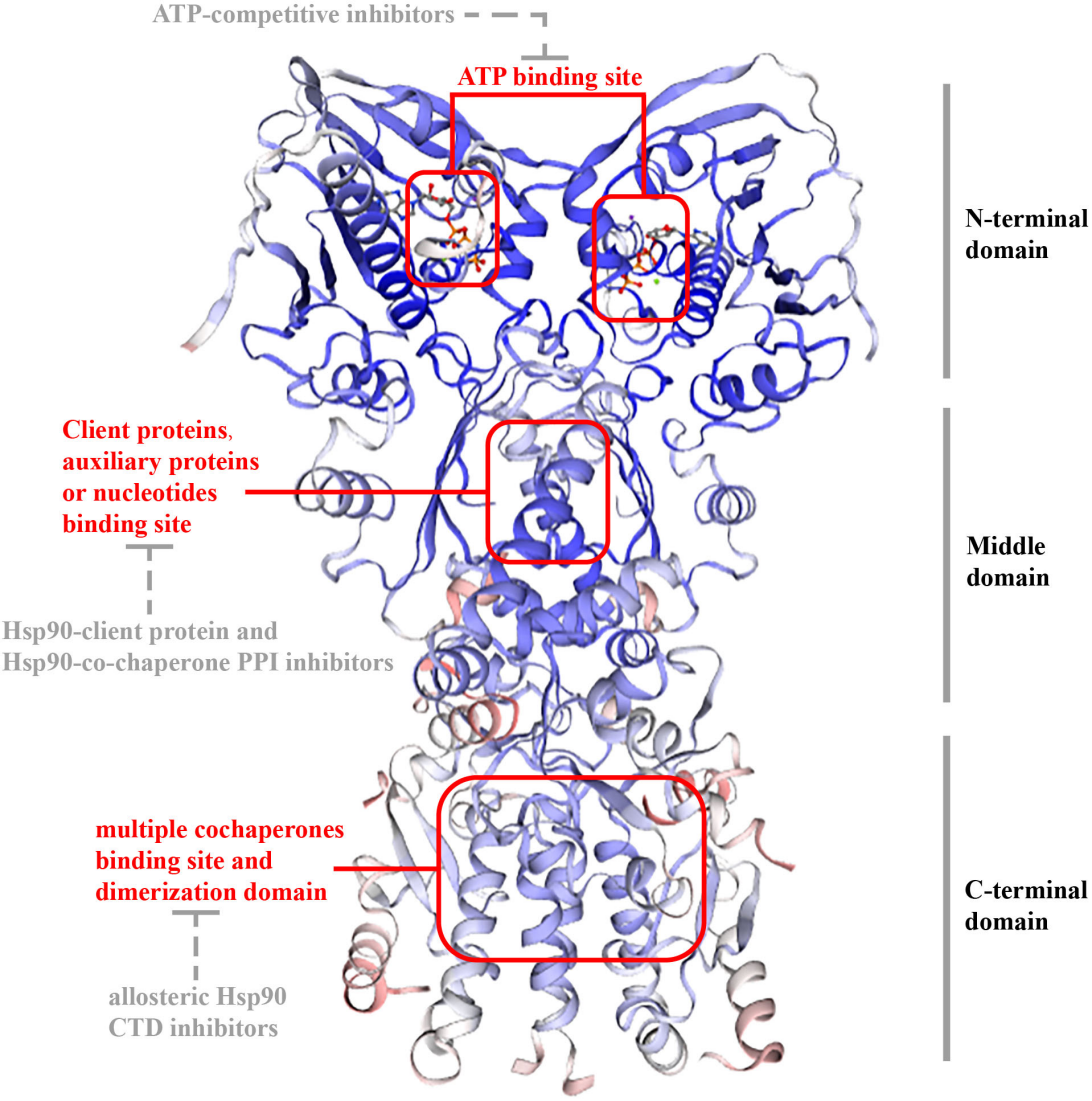
Structure of TRAP1. The structure of TRAP1 is similar as other HSP90 proteins (Hsp90α, Hsp90β, and Grp94): 1) they have three sections: NTD; MD; CTD. 2) they have an ATP-binding site in a deep pocket on the helical face of NTD, targeted by ATP-competitive inhibitors; and client protein binding sites in MD for interplaying with some client proteins and auxiliary proteins or nucleotides, targeted by Hsp90-client protein and Hsp90-co-chaperone PPI inhibitors. CTD is in charge of binding multiple cochaperones and includes dimerization domain, and allosteric Hsp90 CTD inhibitors targets the domain. However, unlike other HSP90 proteins, TRAP1 did not contain a conversed amino acid sequence, which is crucial for interactions with modulators at the end of CTD.

In eukaryotes, Hsp 90 (90 kDa) is a three-domain molecular chaperone protein with a highly conserved biological structure. It contains three domains (**Figure 1**): an N-terminal domain(NTD) includes an ATP-binding site in a deep pocket on its helical face (17); a middle domain (MD), docking some client proteins and auxiliary protein; and a C-terminal domain(CTD), which contains unique motifs MEEVD or KDEL, depending on its isoform and cellular location and is in charge of binding multiple cochaperones and includes dimerization domain (8, 18). There are also certain inhibitors for each domain. ATP-competitive inhibitors targets NTD, Hsp90-client protein and Hsp90-co-chaperone PPI inhibitors targets MD, while allosteric Hsp90 CTD inhibitors targets CTD (19).

However, in comparison with Hsp90 (or Grp94), the first 59 amino acids in NTD of TRAP1, a mitochondrial targeting sequence at NTD that is cleaved after mitochondrial translocation (20–22); TRAP1 also lacks a highly charged elastic region (CR) between the NTD and MD for regulating chaperone function (23, 24). Among other Hsp90 families, the N-terminal domain contains highly conserved amino acid sequences, such as the MEEVD (Met-Glu-Glu-Val-Asp) sequence, that can interact with other proteins. Nevertheless, TRAP1 does not have this sequence, indicating it has no cochaperones (25). Besides, TRAP1 and bacterial chaperone high-temperature protein G (HtpG) can form tetramers as a dimer of dimers, which are related to mitochondrial metabolic regulation (26).

The structure of TRAP1 is not as same as other HSP90 proteins, but the interaction mechanisms with other client proteins are varied. Hsp90 must extensively interplay with cochaperones to coordinate their cycle and recruit clients following the structural changes between open and closed conformations (27, 28). TRAP1 acts as a similar conversed mechanism, but the TRAP1 cochaperones have yet to be reported. Moreover, unlike other Hsp90 proteins, TRAP1 does not need steroid receptors to support signaling protein folding and maturation (28). The amphipathic helix section in Hsp90, which contains E466R, W467R, N470D, M546T, M550A, L553A and F554A, is a client interacting region (29). Furthermore, the clients likely directly interact with open-conformation TRAP1, and the interplay can maintain stably even without ATP contribution (30–32). Therefore, TRAP1 has a certain degree of autonomy. The clients may load into the open-form TRAP1 through a protein-protein way, leading to ATP hydrolysis, changing the conformation, and enhancing the TRAP1 activity (25).

### 2.2 Mechanism of interaction with client proteins

In comparison to other chaperone protein-requiring Hsp90 (28), TRAP1 is unable to form complexes with known cytoplasmic Hsp90 co-chaperones or to promote maturation of Hsp90 client proteins, suggesting a unique mechanism for TRAP1 (33). The major interactors of TRAP1 are the mitochondrial chaperones mtHSP70 and HSP60 (26). Furthermore, the TRAP1-client interaction itself is unaffected by ATP-mimetic inhibitors that favour the open form and remains stable even in the absence of ATP, indicating that its clients are likely to be loaded by direct interaction with the open conformation TRAP1 (30–32). Mitochondrial contact site and cristae organizing system subunit 60 (MIC60) is a core protein of the mitochondrial cristae, and TRAP1 can directly interact with MIC60 to reduce ubiquitin-dependent degradation in extracellular acidosis. TRAP1 can directly interact with MIC60 to reduce ubiquitin-dependent degradation in extracellular acidosis, thereby attenuating acidosis-induced mitochondrial and cardiac injury (34). Clients load into the open form of TRAP1 through direct protein-protein interactions, triggering the binding and hydrolysis of ATP, which changes the conformation and enhances the chaperone activity of TRAP1 (25). Therefore, TRAP1 is an autonomous molecular chaperone that functions through interactions with clients and ATP.

Generally, TRAP1 has two different chaperone activities, which are called foldase and holdase. The difference lies in the necessity of ATP hydrolysis in chaperone function (35). Foldase-type activation requires ATP hydrolysis, mainly conformationally remodeling clients and stabilizing, activating, or inactivating them. Eventually may help in the formation of large protein complexes. Holdase-type activity functions independently of ATP hydrolysis and primarily regulates the enzymatic activity of clients and in some cases influences the stability of clients (36). Under normal circumstances, TRAP1 functions in the form of dimers, but recently a tetrametric form of TRAP1 was observed in the response of dysregulated oxidative metabolism (26). It is the most stable stoichiometric TRAP1 complex, the level of which varies with decreasing and increasing OXPHOS (26).The foldase activity of TRAP1 enables it to refold unfolded clients with energy from ATP hydrolysis. Based on broad analyses of the structure of TRAP1 binding its client proteins, it was demonstrated that the unfolded clients’ binding surface is usually hydrophobic (37, 38). It was shown that the interaction between TRAP1 and clients is not based on ATP, instead unfolded clients directly bind to a hydrophobic client binding pocket between protomers (30, 31). After clients bind to TRAP1, a conformational transition will be triggered, and TRAP1 turns from an open-form (“apo” state) into a closed state, followed by ATP binding (25). Then an asymmetric homodimeric TRAP1 was formed, in which one protomer was buckled compared to the other. The buckled protomer is better at hydrolyzing ATP, and a subsequent flip in the MD and CTD asymmetry positions the other protomer in the buckled conformation. So, the hydrolysis of two ATP is sequential, and after their hydrolysis, TRAP1 completed the cycle and returned to its symmetric, open state (39). So, in conclusion, it was presumed that the loading of the client triggers the binding of ATP and enhances the ATPase activity of TRAP1 (40). Sequential hydrolysis of two ATPs on TRAP1 helps to remodel the client conformation to release folded or activated clients (25, 41).

**Figure 2:**
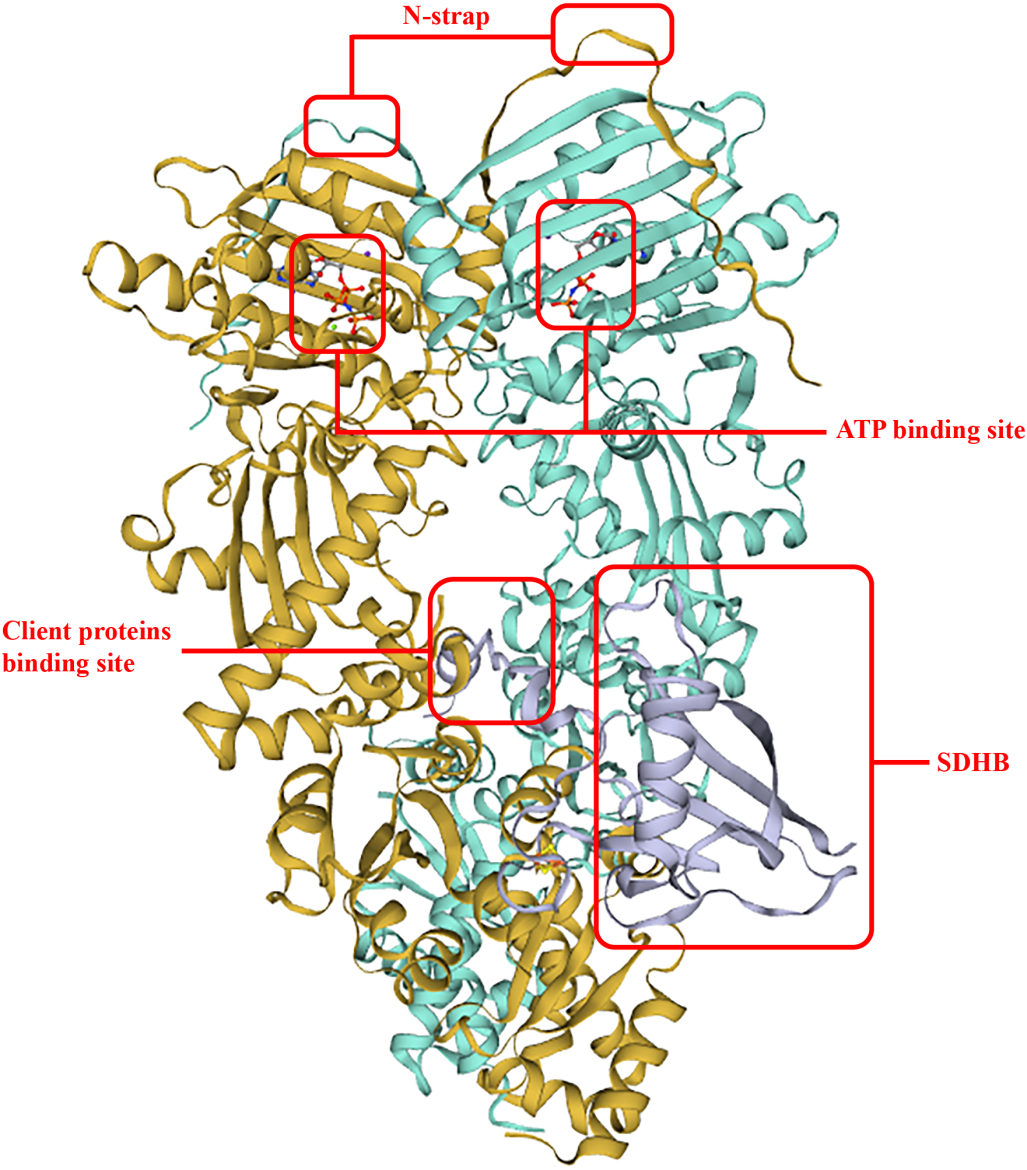
TRAP1 binding with SDHB. The two interplay sites between TRAP1 and SDHB allocates in F531 and F353, respectively. This structure improved ATP hydrolysis and ATP activity, and the binding surface of an unfolded client is conversed and hydrophobic.

To date, the first TRAP1 complex structure with SDHB (B subunit of succinate dehydrogenase) (**Figure 2**) has been detected (PDB: 7KCM) by cyro-EM (33). In detail, an unfolded SDHB segment interacted with the nonpolar residues located in the TRAP1 MD, such as F531 and F353, and was accommodated into a lumen generated between protomers (25). Actually, F353A and F531A played important roles in the interaction of TRAP1 and other clients, and mutations of these residues would reduce binding and compromise the chaperone functions of TRAP1 (32). Moreover, as a post-translational modification of TRAP1, deletion of TRAP1O-GlcNAcylation impairs TRAP1 binding to ATP and TRAP1 interaction with succinate dehydrogenase (SDHB) (42).

Contrary to the foldase activity, TRAP1 exerts its holdase function in other cases, which means that TRAP1 may not induce client folding, but stabilized clients by binding. In this case, TRAP1 may not need its ATPase activity (26). For example, the major mitochondrial deacetylating enzyme sirtuin-3 (SIRT3) showed increased enzyme activity after binding to TRAP1 in the absence of ATP (30). It is worth noting that SIRT3 binding also increases the ATPase activity of TRAP1, which may subsequently (after the holdase function) operate toward SIRT3 and activate it. Therefore, we can conclude that the holdase and the foldase activities likely operate sequentially or synergetically (25).

### 2.3 Mechanism of interaction with inhibitors

Tumors are ubiquitously dependent or “addictive” to a heightened protein folding environment (43), which is vital in buffering the proteotoxic stress accompanying *in vivo* tumor growth (44). Drugs inhibiting or antagonizing mitochondrial Hsp90 and its homolog TRAP1 may have great potential and specificity in treating cancer. Currently, there are several strategies targeting TRAP1. TRAP1 N terminal domain (NTD) inhibition is one of them. NTD is responsible for nucleotide binding and ATPase activity (45). Most inhibitors that have undergone clinical trials as anticancer drugs target the NTD and bind competitively to its ATP binding site to date (46), and representative drugs are Geldanamycin derivatives, guanidine derivatives, and benzamide derivatives. They function by binding the polar residue Asn171 in the middle of the helix of TRAP1 NTD, which locates in the ATP-lid and rotates in to drug binding site (25, 47). Then the foldase activity of TRAP1 is disrupted. Sheperdin is another classical drug targeting TRAP1 by interacting with the ATP-binding pocket in NTD and has cell-penetrating properties (48). However, these competitive inhibitors did not show satisfactory effect *in vivo*, which may be due to their insufficient concentration in mitochondria (5). Some improvements have been made, like conjugating mitochondrion-targeting moieties with ATP-competitive inhibitors or increasing mitochondrial permeability (49). SMTIN is one of those drugs. It contains a triphenylphosphonium (TPP) vehicle that targets mitochondria. SMTIN-C10 (a derivative of SMTIN containing C20 linker) was shown to interact strongly with the ATP binding pocket of TRAP1, while its TPP moiety spread out to interact with allosteric sites in the NTD and MD near the ATP binding pocket, enhancing the antitumor activity (31). Electron microscope structural analysis also found that SMTIN-C10 interaction with TRAP1 induced TRAP1 to a tightly packed closed form (31). Another hotspot ATP-mimic inhibitor under research is gamitrinib-TTP (GA mitochondrial matrix inhibitor-TTP), which has shown feasibility and safety in preclinical trials (50), and phase I clinical trial is ongoing(NCT04827810).

As discussed above, the holdase activity of TRAP1 is independent of ATP binding, and client binding was shown to increase ATPase activity (30). Thus, the holdase activity cannot be inhibited by ATP-binding pocket-targeted inhibitors, and these inhibitors become ineffective in blocking the chaperone function of TRAP1 when it binds to certain client proteins. In these circumstances, the holdase function primarily mediating TRAP-client interaction appears to be essential for the chaperone mechanism. As a result, holdase inhibitors were developed to solve the problem. MitoQ mimics the client and binds to the client-binding site of TRAP1, inhibiting other clients to interact with the chaperone. F353 and F531 residues were shown to participate in the interaction. Although the ATPase activity of TRAP1 is elevated through MitoQ binding, its chaperone function could be fully blocked as other clients were replaced by MitoQ (25).

In addition to the several relatively mature TRAP1 inhibitors mentioned above, many novel compounds have been synthesized and developed with favorable outcomes in recent years. Recently, Merfeld et al. showed a compound 36 that is more than 250 times more selective for TRAP1 than Grp94. In addition, it inhibits OXPHOS, alters cellular metabolism toward glycolysis, destabilizes TRAP1 tetramers, and disrupts mitochondrial membrane potential (51). Kim et al. synthesized a series of pyrazolo[3,4-d] pyrimidine derivatives, in which X-ray diffraction results showed that compounds 47 and 48 interacted with the ATP-binding pocket of TRAP1 protein, and they were shown to exhibit excellent anticancer efficacy in various cancer cells. Moreover, 47 and 48 significantly reduced tumor growth in a mouse PC3 xenograft model (52). Yang et al. developed a series of purine-8-one and pyrrolo[2,3d] pyrimidine derivatives based on the TRAP1 structure, of which 5f was 65-fold more selective than Hsp90α and 13-fold more selective than Grp94, and 6f was 78-fold and 30-fold more selective than Hsp90α and Grp94, respectively (47). The lead compound synthesized by Sanchez-Martin et al. inhibits TRAP1, but not HSP90 ATPase activity, demonstrating the efficacy of using conformational dynamics to expand the chemical space of molecular chaperone antagonists to TRAP1-specific inhibitors with broad therapeutic opportunities (53).

## 3 The functions of mtHsp90 in tumor cell

MtHsp90, including Hsp90 and its homologue TRAP1 were abundantly present in the mitochondria of tumor cells (14). TRAP1 performs different functions in different cancer cell types (54). It can act as a proto-oncogene or an oncogene (15). It is highly expressed in hepatocellular carcinoma (HCC) (55), prostate cancer (56), small cell lung cancer (57, 58), nasopharyngeal carcinoma (59), high-grade glioma (60), and breast cancer (61), but it acts as a tumour suppressor in patients with cervical, bladder, and clear cell renal cell carcinomas (62).

**Figure 3:**
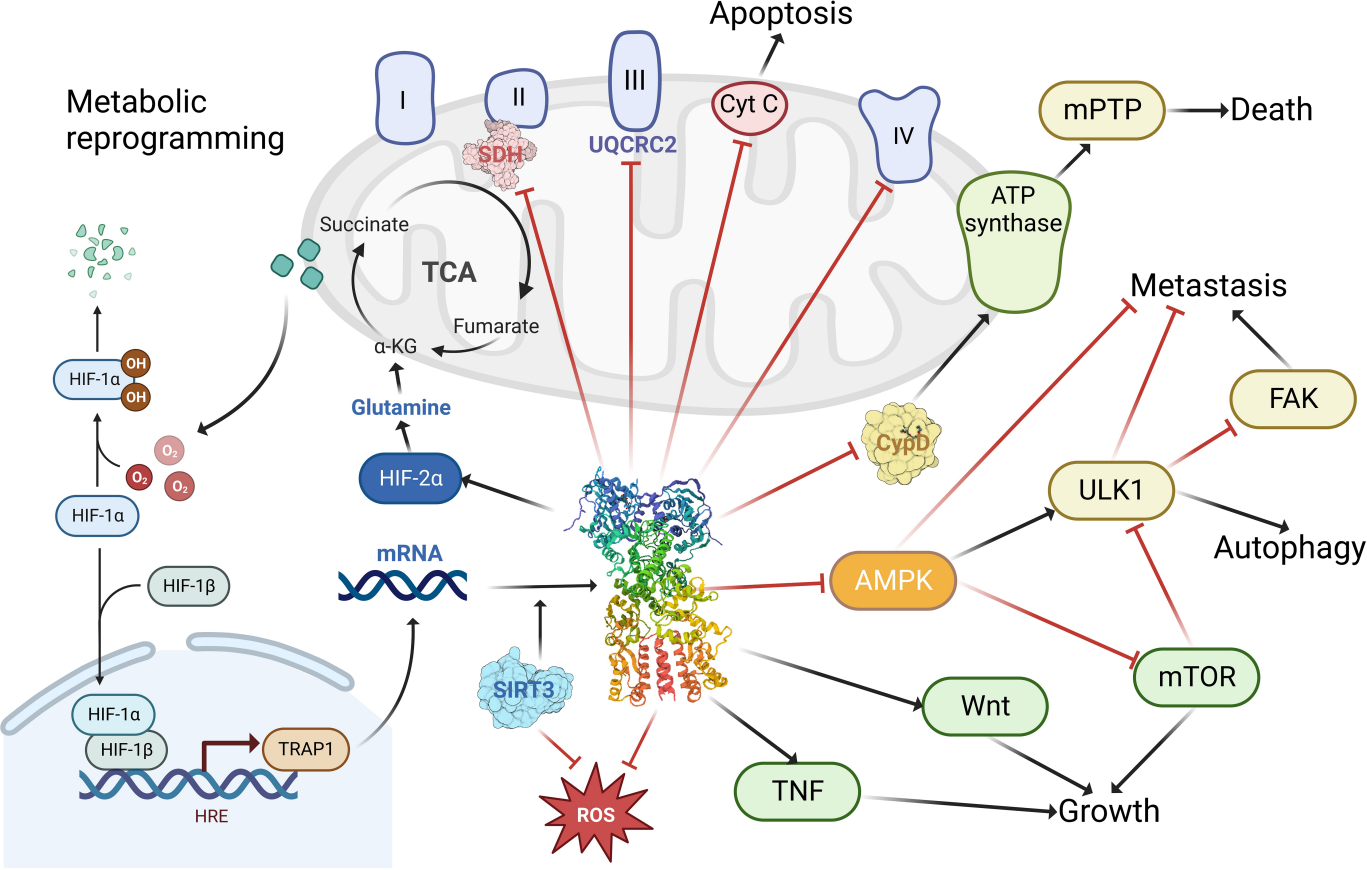
The functions of TRAP1 in tumor cells. TRAP1 binds to and inhibits the activity of complex II succinate dehydrogenase (SDH), complex III core component UQCRC2; enhances cellular utilization of glutamine via HIF-2α; promotes tumorigenesis by stabilizing HIF-1α via succinate; facilitates tumor growth and maintenance through stimulation of the TNF, Wnt pathway; promoting tumor metastasis by activating FAK and inhibiting AMPK and ULK1; modulating apoptosis and autophagy by regulating the release of cytochrome c (Cyt C) and the binding of cyclophilin (CypD) to ATP synthase; blocking ROS generation to prevent cellular damage from oxidative stress. Created with BioRender.com.

TRAP1 is indispensable in the performance of mitochondrial functions and maintenance of structure (**Figure 3**). It has been suggested that TRAP1 plays an essential role in reprogramming the mitochondrial pathway to critically support tumor growth (63, 64). It interacts with key mitochondrial enzymes (32) and has important roles in shaping mitochondrial function, protecting against apoptosis (14) and oxidative stress (65) to critically support cancer cells. It was also shown to control mitochondrial protein quality (66). Specifically, TRAP1 binds mitochondrial and cytoplasmic ribosomes as well as translation elongation factors and facilitates localized translation in the vicinity of mitochondria (67). Moreover, it has pivotal function in mitochondrial homeostasis and bioenergetics (68). The downregulation or depletion of TRAP1 in cancer cells impairs ATP generation and mitochondrial membrane potential, disturbs mitochondrial function (69), reduce proliferation, and has variable effects on apoptosis (57). Not only dose TRAP1 act on mitochondria, but it also participates in lysosomal-mitochondrial crosstalk to maintain cellular homeostasis, and its activation improves the lysosomal phenotype in the cells of patients with a variety of lysosomal storage disorders, and may be a potential therapeutic target for a wide range of lysosomal diseases (70).

MtHsp90 regulate and activate several cancer-related pathways including metabolic reprogramming (71), tumor cell motility, and evasion of apoptosis. These effects enhance tumor cells growth in low nutrients environment and metastatic dissemination to other body parts, also arm them with drug resistance. MtHsp90 was suspected to block drug-induced apoptosis in several tumors (57), conferring tumor cells resistance against chemotherapeutic agents (72).

One of the major functions of mtHsp90 is that it played essential role in tumor cells metabolism reprogramming, which is an important driver of tumor adaptation, generation, and progression. One of the hallmarks of tumor cells in energy metabolism is aerobic glycolysis, which is also called Warburg effect. MtHsp90 is important in this pathway. TRAP1 was suggested to inhibit succinate dehydrogenase (SDH) activity to favor aerobic glycolysis (Warburg effect) and inhibit oxidative phosphorylation (OXPHOS) in human colon, cervix, ovary and bone cancers (62, 73). Sciacovelli et al. (74) demonstrated that TRAP1 binds to and inhibits respiratory chain complex II succinate dehydrogenase (SDH). The respiratory downregulation induced by the interaction of TRAP1 with SDH promotes tumorigenesis by initiating the stabilization of the succinate-dependent oncogenic transcription factor HIF1α. Current TRAP1 inhibitors targeting this pathway have achieved promising results in human cells. The study by Sanchez-Martin et al. demonstrated that honokiol DCA (HDCA) (a small molecule antitumor agent) binds to a variant site in TRAP1 and is able to inhibit TRAP1 but not Hsp90 ATPase activity (75). In Human plexiform neurofibroma 95.6 cells, HDCA restored TRAP1-dependent SDH down-regulation, decreased proliferation rate, increased mitochondrial superoxide levels and eliminated tumorigenic growth (75). Despite the facts mentioned above, a recent study demonstrated that TRAP1 binds the core component of complex III, UQCRC2, within the mitochondria and regulates complex III activity. This reduces respiration rates under basal conditions, but maintains sustained oxidative phosphorylation during glucose limitation (76). Moreover, TRAP1 interacts with PINK1(*PTEN induced putative kinase 1*) to protect cells from oxidative stress-induced cell death (77).

But it was verified to be insufficient to explain the complexity of metabolic reprogramming in tumor cells (78, 79). There must be other metabolism pathways to support high energy demanding of tumors, such as metastasis and cell proliferation, and that’s where mtHsp90 also come into play (68). Under unfavorable nutrient conditions such as nutrient deprivation or amino acid shortage, tumor cells are able to utilized mtHsp90-directed and TRAP1 directed protein folding (14) to maintain ATP production (6), thus attenuating the activation of the nutrient-sensing, tumor-suppressive AMPK pathway (80) and tumor cell autophagy pathway (6). Tumor cells are conferred with a survival and proliferation advantages and their progression and invasion under nutrient deprivation was thus possible then (71). Overexpression of TRAP1 in cancer associated fibroblasts (CAFs) increases basal oxygen consumption rate (OCR) and ATP production. *In vivo*, overexpression of TRAP1 in CAFs of HSC3 cell xenografts model inhibited tumor growth (81). The mechanisms behind maintained ATP production were the retention of hexokinase II to the mitochondrial outer membrane by regulating cyclophilin D (CypD) folding (6), keeping the stability of oxidative phosphorylation complex II subunit (79) and succinate dehydrogenase. In conclusion, mtHsp90 mediated energetics can maintain a residual level of oxidative phosphorylation to block the activation of tumor-suppressive AMPK signaling and autophagy (6), which are considered vital for tumor progression and drug resistance *in vivo*. Although the promotion effects of TRAP1 in Warburg effect and oxidative phosphorylation (i.e. inhibit or activate SDH) in cancers seemed to be opposing, it was just a result of different metabolic plasticity under different tumor environments (25). Furthermore, it has been suggested that TRAP1 is essential for malignant transformation of cells but is dispensable in later stages of tumour development (30, 62). Although the function of TRAP1 is controversial, the majority of the literature suggests that TRAP1 is overexpressed in many cancers and regulates metabolic transformation during tumourigenesis, and that TRAP1 attenuation is detrimental to tumor cell survival (16, 61, 82–85). The ultimate function of TRAP1 in pathology is still tumorigenesis. The cellular contexts affecting these contradictory effects are still poorly understood, but upstream regulators and clients of TRAP1 may be one of them, such as the bi-directional interaction between SIRT3 (a NAD^+^-dependent deacetylating enzyme) and TRAP1 (25).

MtHsp90 also promote tumor cell motility and metastasis *in vivo*, especially in case of scarce nutrients. That may explain why these chaperones are ubiquitously overexpressed in advanced tumors. Recent research has found that TRAP1 overexpression (TRAP1 OE) promotes mitochondrial fission, enhanced *in vitro* migration and *in vivo* metastasis of tumor cells, and altered cellular homing properties (86). In a study (71), researchers use noncytotoxic concentrations of gamitrinib to antagonize the function of Hsp90 in mitochondria. The result showed a nearly complete suppression of invasive length and areas of tumor cells. Next, they use pooled siRNAs to silence TRAP1, the same effect was reproduced, demonstrating mtHsp90 ‘s importance in tumor cell motility. Through further investigation, they found that tumor cell motility was also influenced by cell motility kinases, tumor cell bioenergetics and tumor cell autophagy. These pathways were regulated by mtHsp90 to preserve cytoskeletal dynamics through continuous phosphorylation of multiple cell motility kinases and releasing FAK from inhibition by ULK1-FIP200 autophagy-initiating complex (87). All the above contribute to tumor cell invasion and dissemination, leading to shorted OS in patients. On the contrary, activation of AMPK, ULK1 or autophagy regulators suppressed tumor progression and restricted motility (71). In conclusion, mtHsp90 regulate the crosstalk between bioenergetics stress, AMPK activation and autophagy to preserve tumor cell motility.

MtHsp90 also prevents autophagy, another tumor-suppression mechanism, in cancer cells during nutrient deprivation (71, 88) to preserve tumor cell motility. Inhibition of TRAP1 in tumor cells caused marked increase in lysosome content and autophagy activity (69).

Cancer cells’ evasion of apoptosis is mediated partly by mtHsp90, too. TRAP1 improve cancer cells’ resistance to various stresses including anticancer therapies by upregulating cell death threshold (89, 90). TRAP1 can protect mitochondria against reactive oxygen species (ROS) and oxidative stress (64) by blocking ROS production (91)(e.g. suppress SDH activity) or scavenging noxious ROS (92)(e.g. activate superoxide dismutase 2 through SIRT3 (30)). There are also other anti-death mechanisms aside from scavenging ROS. For example, it inhibited the release of cytochrome c (64). It also antagonized cyclophilin D (CypD) (93) to inhibit the opening of CypD-dependent mitochondrial permeability transition pores (mPTP) (94, 95), and mPTP opening is directly related to cell death. Upon cellular stresses, CypD was shown to irreversibly open mPTP by changing the conformation of complex V, which is one of the components of mPTP (96). So TRAP1 inhibit CypD and preserve the integrity and membrane potential of mitochondria, which is a vital survival mechanism for tumor cells (63). TRAP1 also ameliorates diabetes-induced kidney injury by preventing abnormal opening of mPTP and maintaining mitochondrial structure and function (97).MtHsp90 was reported to interact with CypD to antagonize mitochondrial permeability transition process, too (14). It was also showed to be associated with enhanced DNA repair when patients were treated with cisplatin (98). A study by Zhang et al. in 2021 demonstrated that TRAP1 inhibits cisplatin-induced apoptosis by promoting ROS-dependent mitochondrial dysfunction (99). Combined low activity of DNase1 and Trap1 results in impaired chromatin degradation *in vitro*, delayed chromatin clearance *in vivo*, and enhanced immune cell activation (100). Expression of TRAP1 and HSPD1 correlates with DNA replication and mitotic inhibitor sensitivity (101).

However, cancer cells differ from normal cells in many aspects, including altered expression levels and functional states of mitochondrial Hsp90 and TRAP1 (102, 103). Many cancer cells express higher levels of mitochondrial Hsp90 and TRAP1, up-regulate the associated signaling pathways and depend on them for survival, (e.g. TRAP1 promotes neoplastic growth through inhibiting succinate dehydrogenase (104)) making them vulnerable to drugs that inhibit these molecules, normal cells have lower expression levels of mtHsp90, thus less susceptible to drugs targeting mtHsp90. Additionally, some targeted drugs have good tissue distribution and pharmacokinetic properties, which allow them to enter tumor tissues and exert their effects more easily (105). MtHsp90 inhibitors have high affinity and specificity for cancer cells, and the reason may lie in the unique chaperone level, localization (69) and metabolism of cancer cells compared to normal cells. Tumor cells rely far more on Hsp90 than normal cells, leading to their vulnerability to Hsp90 inhibitors. TRAP1 is even more special. It functions only in response to cellular stress instead of maintains housekeeping protein homeostasis, and when this stress-adaptive machinery goes dysregulated, human diseases, such as cancer, may arise (25). TRAP1 is highly expressed in tumors such as glioblastoma, colon, breast, prostate and lung cancers (106), but is low or even undetectable in normal cells (64). As a result, TRAP1 deletion was shown to have no or minimal effect on normal development of mice (68), but can effectively inhibit growth of tumor cells. In another study, TRAP1-depleted tumor cells exhibited decreased cell viability, but the same negative impact of TRAP1 depletion was not observed in normal cells (69). For example, glutamine metabolism is dysregulated in various types of cancers, which are termed “glutamine auxotrophic cancer”. They became to rely mainly on glutamine-dependent metabolism for ATP production, and the process was regulated by TRAP1. Dharaskar et al. in a recent research demonstrated that in tumor cells TRAP1 maintains mitochondrial integrity during glucose deprivation and enhances cellular utilization of glutamine for cellular energy requirements via the HIF2α-SLC1A5-GLS axis (107). So TRAP1 inhibition will only influence these addictive cells, posing no threats to normal cells, i.e. glutamine-independent cells (108). Therefore, cancer cells show differential sensitivity to Hsp90 inhibition based on drug metabolism compared with normal cells (109).

In addition to its significant role in the development, metastasis, and maintenance of cancer, aberrant expression of TRAP1 has been associated with a number of diseases, including but not limited to the following: TRAP1 overexpression protects motor neurons from mitochondrial dysfunction and death under conditions of oxidative stress caused by amyotrophic lateral sclerosis (ALS) (110). Ramos Rego et al. describe the role of TRAP1 in CNS cells under physiological and pathological conditions (111). TRAP1 regulates hypoxia-induced cardiomyocyte apoptosis through the COXII-mediated mitochondria-dependent apoptotic pathway (112). And in the human renal proximal tubular epithelial cell line HK2, Lin et al. found that TRAP1 is important for the maintenance of mitochondrial function in HK2 cells under pathological conditions and the activation of TRAP1 may be useful in the treatment of renal fibrosis (113).

## 4 MtHsp90 inhibitors in cancers

Compared to other Hsp90 inhibitors, many new small molecule drugs that target TRAP1 or mitochondria show better performance in controlling cancer cells and reducing cellular toxicity. Extensive safety and effectiveness tests *in vitro* and *in vivo* have been conducted, further demonstrating the potential of mtHsp90 inhibitors in cancer treatment.

### 4.1 Lung cancer

Lung cancer is one of the most common types of cancer and is responsible for the highest number of cancer-related deaths worldwide. It is a malignant tumor that starts in the cells of the lungs and can quickly spread to other parts of the body. The non-small cell lung cancer (NSCLC) is the most common type of lung cancer, accounting for approximately 85% of all cases. The advent of tyrosine kinase inhibitors (TKIs) has profoundly impacted the clinical outcomes of patients with NSCLC that exhibit EGFR-activating mutations, which is one of the most common contributing factors in NSCLC (114, 115). However, despite the initial success of EGFR TKIs, resistance eventually develops in many patients (116). Moreover, patients with NSCLC who harbor other mutation types, such as KRAS mutation, still lack effective treatment options and are more susceptible to developing resistance for current therapies (117). Drug resistance always arises from the evolutionary pressure exerted on NSCLC cancer cells by TKIs, which leads to insensitivity and failure in clinical treatment. Therefore, to induce this drug resistance of TKIs, many different methods have been tried and some of them showed a prospective result for NSCLC control, such as the combined EGFR/Hsp90 inhibition (118).

This special advantage of mtHsp90 inhibitors has been evaluated in many studies and demonstrated its ability to control the development of NSCLC. Tumors collected from Gamitinib-treated animals showed extensive apoptosis *in situ* and release of cytochrome C in the cytoplasm, while organs collected from non-tumor regions showed no significant changes in histology, no change in overall structure, and no evidence of inflammation or hepatic steatosis (119). In another study focused on NSCLC, G-TPP has been shown to increase glutamine synthetase activity and induce cell death in glutamine-dependent NSCLC cells by causing an energy shortage, as evidenced by the phosphorylation of AMP-activated protein kinase (AMPK) (108). Furthermore, due to the specific mitochondrial localisation of TRAP1, TRAP1 selectivity at the cellular level can be achieved by attaching mitochondria-targeted portions to pan Hsp90 inhibitors. Gamitrinib- triphenylphosphonium (Gamitrinib-TTP) and SMTIN-P01 were demonstrated to have cytotoxic effects associated with mitochondrial membrane depolarisation in the lung adenocarcinoma cell line H460 (5, 119). And they also had no effect on Hsp90 client protein levels, suggesting its TRAP1 selectivity. Moreover, Hsp90 inhibitor-mitochondria targeting indocyanine dye conjugate (IR-PU) has high apoptosis-mediated cytotoxicity in the NCI-H460 cancer cell line (120).

Preclinical studies in cell lines and animal models have shown promising results for the use of mtHsp90 inhibitors in NSCLC treatment.

These findings also suggest that combining mtHsp90 inhibitors with other drugs targeting tumor-related pathways may be an option to improve treatment outcomes for NSCLC patients in the future. Recently, gamitrinib-TPP has successfully completed preclinical evaluation and is currently in a Phase I clinical trial in advanced cancer. This study was last updated on February 8, 2023, and it is estimated to be completed initially in December 2028 (ClinicalTrials.gov NCT04827810) (121). Additionally, A recently published article reveals the mechanism of TRAP1-associated cancer treatment with CVM-1118 (foslinanib), a phosphoric ester compound selected from 2phenyl-4-quinolone derivatives. In the NSCLC cell line A549, CVM-1125 reduces cytosolic succinate levels and causes destabilization of HIF-1α by targeting TRAP1, thereby blocking its downstream signaling and ultimately inducing mitochondrial apoptosis, inhibiting tumor cell growth, and suppressing angiogenic mimicry (122). The drug has completed clinical Phase I (NCT04336124) and is currently recruiting for clinical Phase II (NCT05257590), with an expected primary completion date of September 2025.

### 4.2 Prostate cancer

Prostate cancer is a prevalent cancer type among men, with over 1.4 million cases diagnosed worldwide annually, which is the second most common solid tumor in men and the fifth cause of cancer mortality over the world (123, 124). It originates in the prostate gland, a small walnut-shaped organ responsible for the production of seminal fluid in males. In its initial stages, prostate cancer frequently manifests no symptoms, but as the cancer progresses and metastasizes, it can cause a range of symptoms, including urinary difficulty, dysuria, hematuria, hematospermia, and pain in the lower back, hips, or thighs (125). Lisanti et al. (82) demonstrated that TRAP1 increases cell proliferation, decreases apoptosis, and promotes cell invasion in prostate cancer without changes in mitochondrial bioenergetics through a common altered Pten +/- context in human prostate cancer.

In the prostate tumor, the mtHsp90 homologous TRAP1 is substantially and differentially expressed in human local and metastatic prostate cancer. In contrast, TRAP-1 is largely undetectable or poorly expressed in normal prostate (56). The treatment of Gamitinib can result in the rapid and complete killing of androgen-dependent or non-androgen-dependent prostate cancer cell types without affecting untransformed prostate epithelial cells. With the loss of organelle membrane potential, release of cytochrome c, and caspase activity, gamitrinib can induce acute mitochondrial dysfunction and then lead to apoptosis in prostate cancer cells (126). In the study of Kang et al., the systemic administration of gamitrinib-G4 was shown inhibition effects on the development of tumor cells in mice with localised and metastatic prostate cancer (127). Furthermore, they found no significant animal weight loss or organ toxicity was observed in response to the continuous gamitrinib-G4 treatment, which confirmed the safety and feasibility of the mtHsp90 inhibitors *in vivo*.

While the results of these preclinical studies are promising, there is still much more research that needs to be done to fully evaluate the potential of mtHsp90 inhibitors for the treatment of prostate cancer in the future. One of the biggest challenges is identifying the most appropriate patient populations and treatment regimens for these inhibitors. Moreover, it is necessary to carefully evaluate the potential adverse events of mtHsp90 inhibitors in humans, as there still is a lack of clinical trial results. However, despite these challenges, the development of mtHsp90 inhibitors represents an exciting new approach to the treatment of prostate cancer in the future. By targeting a critical protein involved in the survival and growth of prostate cancer cells, these inhibitors have the potential to offer a new and effective treatment option for patients with advanced prostate cancer.

### 4.3 Glioblastoma

Glioblastoma is an aggressive form of brain cancer that arises from the glial cells that support the neurons in the brain. It is the most common primary brain tumor in adults, accounting for approximately half of all malignant brain tumors (128). Despite recent advances in multimodal therapy for glioblastoma, including surgery, radiation therapy, and immunotherapy, the overall prognosis remains poor, with a 5-year survival rate of less than 5% and a median survival of fewer than 2 years, and there is currently no effective drug to prolong median overall survival or control recurrence (129, 130). However, the use of mtHsp90 inhibitors may be one promising area of research in glioblastoma, as it will influence lots of tumor-related pathways.

TRAP1 is a major chaperone of the respiratory complex of the electron transport chain, therefore interfering with TRAP1 disintegrates oxidative phosphorylation (131). Besides, TRAP1 is upregulated in Glioblastoma multiforme (GBM) compared with normal brain cells. The high expression of TRAP1 can affect the cell glycolysis in the tumor microenvironment (132). TRAP1 might take a role in maintaining the stemness of glioblastoma stem cells. TRAP1 protects mitochondrial integrity and prevents apoptosis (133), thus induces the resistance to temozolomide (TMZ), the standard chemotherapy drug for glioma. Therefore, TRAP1 is a promising target for drug design in glioblastoma therapy (134).

Gamitrinib has been reported in glioblastoma that through suppressing TRAP1, gamitrinib can sensitize the GBM cells to temozolomide treatment (134). It inhibits cell proliferation and induces apoptosis and death in 17 primary glioma cell lines, 6 TMZ-resistant glioma cell lines, 4 neurospheres and 3 PDOs (135).Another research showed that by inducing mtUPR and the subsequent ROS burst, TRAP1 function was inhibited and GBM cells were sensitised to TMZ lysis after gamitrinib treatment (136). Moreover, in cell line–derived xenografts and patient-derived xenografts models implanted with subcutaneous or intracranial tumors, gamitrinib could also significantly delay tumor growth and increased mouse survival. Through integrated analysis of RNAseq and RPPA data, they revealed that gamitrinib exhibited anti-tumor activity mainly by (i) inhibiting mitochondrial biosynthesis, OXPHOS, and cell cycle progression, and (ii) activating energy-sensing AMP-activated protein kinase, DNA damage, and stress response. These preclinical findings provide robust and convincing evidence that supports the potential therapeutic efficacy of gamitrinib in the management and treatment of neuroglial tumors.

Combination therapy has also been studied in glioblastoma. Combining LXR agonist and TRAP1 inhibitor gamitrinib-TPP resulted in increased levels of Bcl-2 family protein expression and higher rates of cell death (136). In addition, the combined use of both gamitrinib and histone deacetylases (HDAC1/2) inhibitors (e.g., Romidepsin or Panobinostat) can further reduce tumor growth in model systems of glioblastoma (137). In murine model systems of patient-derived orthotopic xenografts of human glioblastoma (PDX), the combination of BH3-mimetics and gamitrinib-TPP blunted cellular proliferation in a synergistic manner by massive activation of intrinsic apoptosis (138). These preclinical findings indicate the potential of gamitrinib in treating glioblastoma.

### 4.4 Breast cancer

Breast cancer is a complex and heterogeneous disease that affects millions of women worldwide. Breast cancer is estimated to be the most common cancer overall and the top 2 cause of death in women (123). It is a type of cancer that begins in the breast tissue and can spread to other parts of the body. Despite the relatively favorable prognosis and advances in the treatment of breast cancer, there is still a need for more effective and targeted therapies for different cancer subtypes. The use of mtHsp90 inhibitors in the treatment of breast cancer may be one of the promising ways.

Liu et al. suggested that TRAP1 expression promotes cell proliferation and tumor growth through the TNF pathway, while its downregulation may lead to reduced proliferation and increased metastatic potential (139). An Aberrant upregulation of TRAP1 has been reported in the tumorigenesis of breast cancer (61). Different from other carsinoma, it has nothing to do with the proliferative capacity of cancer cell (140). Instead, TRAP1 modulates mitochondrial dynamics and function, and links these processes to the tumorigenesis of breast cancer. When TRAP1 is overexpressed, the mitochondria will be less fragmented and more tubular network-shaped and the mitochondrial aerobic respiratory will be upregulated. This suggests that TRAP1 may be a potential target for breast cancer therapy (61).

Several mtHsp90 inhibitors have been developed and shown to have promising activity in preclinical models of breast cancer. For example, gamitrinib has been shown to induce apoptosis in breast cancer cell lines and to inhibit the growth and spread of tumors in mouse models of the disease (119). Gamitrinib-TPP, an inhibitor targeting mtHsp90, increases cell death in MCF7 cells (141). Similarly, SMTIN-P01 has been shown to selectively accumulate in the mitochondria of breast cancer cells and induce cell death (5). However, although mtHsp90 inhibitors demonstrate significant advantages in controlling the development of breast cancer cells and tolerating side effects, there are still some limitations with the tests in other types of cancer. Further research is still needed to ensure the feasibility of these inhibitors before human testing.

### 4.5 Gastric cancer

In 2020, 769,000 people died from gastric cancer (GC) worldwide (123), ranking as the fourth most common cause of cancer-related deaths. Targeted drugs for gastric cancer have been developed, for instance, Trastuzumab, a monoclonal antibody against HER2. When combined with chemotherapy, it can improve the survival rate of advanced gastric cancer, but the resistance remains to be tackled (142).

Hsp90 plays an important role in gastric carcinogenesis by activating downstream client proteins, including RAF, KIT, EGFR, HER2, etc. (143).TRAP1 is an important member of mtHsp90 that inhibits the survival of reactive oxygen species ROS, thus protecting cells from mitochondrial apoptotic mechanisms and guaranteeing a sustained proliferative state (144, 145).

A study by Ping Han et al. (146). showed that the mRNA and protein expression levels of TRAP1 were significantly higher in cancer tissues than in adjacent normal tissues. In addition, TRAP1 may regulate the malignant biology of cells by increasing the expression of CyclinB1, CyclinD1, CyclinE, MMP-2, and VEGF, leading to the development and progression of gastric cancer, which is an important target for targeted therapy. H. pylori vacuolating cytotoxin A (vacA) is involved in the regulation of apoptosis in human gastric epithelial cells by inducing down-regulation of TRAP1 via the P38MAPK pathway (147). In esophageal cancer (EC) TE-1 cells, shikonin promotes apoptosis and attenuates migration and invasion through inhibition of TRAP1 expression and AKT/mTOR signaling, suggesting that shikonin may be a novel drug for the treatment of EC (148).

Nevertheless, no more targeted drugs are developed for TRAP1 in gastric cancer until now, and the therapeutic field for gastric cancer targeting mtHsp90 remains blank.

### 4.6 Colorectal cancer

Bowel cancer consists of large bowel cancer and small bowel cancer. Most bowel cancer begins in the colon (149). The colon and rectum make up the large intestine. Cancer that starts here is called colorectal cancer (CRC). Colorectal cancer is the second and the third most commonly occurring cancer in women and men, respectively (150), and one of the most common causes of cancer deaths (151).

According to the investigation, in colorectal cancer samples, Hsp90 expression is highly increased compared with normal epithelial tissues. Hsp90 might take a pathological role in colorectal cancer *in vivo* (103). Particularly, in colorectal cancer, over-expression of TRAP1 might encourage tumor cell invasion (152). TRAP1 is up-regulated in ulcerative colitis associated colorectal cancer (102). TRAP1 has been suggested as a predictive marker for prognosis in colorectal cancer (153), human metastatic colorectal carcinoma (104) and ulcerative colitis-associated colorectal cancer (102).

TRAP1 is possibly involved in the regulation of colorectal cancer through a variety of mechanisms. TRAP1 regulates stemness through Wnt/β-catenin pathway in human colorectal carcinoma (154). Upregulated in 60-70% of human colorectal cancers (CRC), TRAP1 regulates the Wnt/β-Catenin pathway and prevents β-Catenin phosphorylation/degradation by modulating the Wnt ligand receptors LRP5 and LRP6, which facilitates stem cell maintenance (155). Condelli report that TRAP1 can participate in the progression of colorectal cancer through regulating the synthesis and ubiquitination of BRAF (156). In human colorectal cancer cells and tissues, with high TRAP1 background, the protein expression and phosphorylation of p70S6K is reduced (157). When colorectal cancer is under oxygen deprivation, TRAP1 regulates the response of cells to hypoxia and inhibits ribosome biogenesis (158). It is involved in regulating hypoxia-induced HIF-1α stabilization and glycolytic metabolism. Moreover, glucose transporter protein expression, glucose uptake and lactate production were partially impaired in TRAP1-silenced CRC cells under hypoxic conditions (158). TRAP1 also cooperate with soluble resistance-related calcium-binding protein (sorcin) in human colorectal carcinoma in a survival pathway, which is responsible for inducing multi-drug resistance (72).

For patients with Hsp90-positive rectal cancer, the application of suitable Hsp90 inhibitors would be highly beneficial (103). Since broad spectrum inhibitors of Hsp90 family have demonstrated negative efficacy on TRAP1 inhibitory effect due to its poor mitochondrial permeability, researchers have paid attention to TRAP1 isoform-selective inhibitors. Shepherdin is the first peptidomimetic with the ability to permeate into the mitochondria and target TRAP1 (105). Novel compounds, for example mitochondrial permeating SMTIN-P01 (64) and the most selective TRAP1 inhibitor DN401 (5) are also developed.

Clinically, Hsp90 inhibitors are often combined with chemotherapy to treat with CRC. A previous study revealed that after combined use of AUY-922 with 5-FU, CRC cells exhibited a lower multi-drug resistance (159). Targeting TRAP1 by gamitrinib induces BRAF-driven apoptosis and inhibits colony formation in CRC cells (160). Gamitrinib-TPP, inhibiting TRAP1 signaling pathways in colon cancer, can disrupt redox homeostasis and induce cell death. Under oxidative stress, inhibition of TRAP1 by gamitrinib-TPP induced metabolic reprogramming and heat shock factor 1 (HSF1)-dependent transactivation, with enhanced induction of DNA damage and cell death (161). Combining with BH3-mimetics, gamitrinib-TPP actives intrinsic apoptosis (138). Moreover, combination of LXR agonists and gamitrinib-TPP induces elevation of pro-apoptotic Bcl-2 family and Noxa in HCT116 cells (136). However, colon cancers can induce variable ER stress responses and ROS accumulation to resist gamitrinib-TPP treatment. Tsai report that treatment with both an NRF2 inhibitor and a TRAP1 inhibitor may potentially overcome colon cancer resistance by raising cellular ROS level (162).

Undoubtedly, targeted drugs are becoming more and more important in the field of clinical oncology. However, considering the cytotoxicity and induced multi-drug resistance, there is still a long way to go before drugs targeting mtHsp90 can take a role in the therapy of colorectal cancer.

### 4.7 Liver cancer

Liver cancer is the third leading cause of cancer-related deaths worldwide. Hepatocellular carcinoma (HCC) is the most common liver cancer, accounting for 90% of liver cancer. It is predicted that 1.3 million people could die from liver cancer in 2040. Traditional treatment methods for liver cancer mainly include: surgery, liver transplantation, ablation therapy, interventional therapy, radiation therapy, and chemotherapy. The new therapies can be mainly divided into the following three categories: targeted drug therapy, immunotherapy, combined treatment strategies, etc. (163).

The study found that the high expression of Heat Shock Protein-90 (Hsp90) was related to the low survival rate of hepatocellular carcinoma (164). Experiments have found that Hsp90 is highly expressed in liver cancer patients (165–168). TRAP1 can regulate mitochondrial integrity, prevent oxidative stress and inhibit cell death (169). Ramos Rego et al. examined the TRAP1 interactome using the tandem affinity purification system and identified 255 unique proteins, which can regulate a variety of cellular processes, including energy metabolism, suggesting that TRAP1 maintains mitochondrial integrity in addition to metabolic rewiring (170).

It is possible that the expression of TRAP1 is associated with autophagy in liver cancer; HepG2 cells exhibited the highest basal level of autophagy and TRAP1 expression with medium invasive ability. Moreover, hepatitis B (HBV) infection of HepG2 cells suppressed autophagy activity and the expression of TRAP1. Treatment with rapamycin also greatly increased autophagy in the 4 liver cancer cell lines and enhanced the expression of TRAP1 in HepG2, Hep3B2.1-7 and Sk-hep1 cells. Therefore, TRAP1 may be related to autophagy in liver cancer, as cell invasiveness, HBV infection and autophagy induction have different effects on TRAP1 expression (171). Another study showed that S-nitrosylation of the mitochondrial chaperone TRAP1 sensitises hepatocellular carcinoma cells to succinate dehydrogenase inhibitors. Chromosomal deletion of GSNOR leads to pathological protein S-nitrosylation implicated in human hepatocellular carcinoma (HCC). This study identifies and exploits a metabolic hallmark of aberrant S-nitrosylation in HCC, demonstrate that GSNOR deficiency in hepatocytes is characterised by mitochondrial alterations and marked increases in levels and activity of succinate dehydrogenase (SDH). This is dependent on selective S-nitrosylation of Cys501 in the mitochondrial chaperone TRAP1, mediating its degradation. As a consequence, cells and tumours which are GSNOR-deficient are extremely sensitive to SDH inhibition, namely to α-tocopheryl succinate, a molecular targeting SDH, which induced RIP1/PARP1-mediated necroptosis and inhibited the growth of the tumour (171).

### 4.8 Leukemia

Leukemia refers to a set of heterogenous hematological malignancies, including acute myeloid leukemia (AML), acute lymphoblastic leukemia (ALL), chronic myeloid leukemia (CML) and chronic lymphocytic leukemia (CLL) (172). It is characterized by the rapid growth and accumulation of abnormal, immature white blood cells (173), which consequently interfere with the production of normal blood cells (174). Treatment may involve chemotherapy, radiotherapy, stem cell transplantation, targeted therapy (175), or a combination of these approaches (176).

Studies have found that Hsp90 is overexpressed in leukemia and its expression has been linked to poor prognosis (177). In particular, TRAP1 is prominently upregulated in pediatric AML patients according to bioinformatics analysis of public databases (178).

The overexpression of TRAP1 can promote neoplastic growth through a variety of mechanisms, including inhibiting succinate dehydrogenase (104) and reducing ROS (179). Therefore, targeting mtHsp90 may be a potential strategy for treating leukemia.

Currently, some drugs targeting at mtHsp90 have been developed. Gamitrinibs (GA mitochondrial matrix inhibitor) is a class of small molecule compound that selectively inhibit the activity of mtHsp90, including gamitrinib-G1–G4 and gamitrinib-TPP (119). Gamitrinibs inhibit AML cells both *in vitro* and *in vivo* preclinical trials. With systemic administration of gamitrinib-G4 to mice, the growth of established human leukemia is inhibited, The lead compound gamitrinib-TPP also shows significant anti-cancer activity against hematologic malignancies (119). Recently, Mathieu et al. designed and synthesized a series of 6BrCaQ-Cn-TPP conjugates as a novel class of TRAP1 inhibitors, among which compound 3a exhibited excellent antiproliferative activity in a variety of cancer cell lines, including human leukemia cells K562 (180).

Overall, there have been several drugs that specifically inhibit mtHsp90. These drugs have shown promising results in leukemia in preclinical studies. However, more research is in need to fully understand the role of these drugs in leukemia and to determine their efficacy and safety.

### 4.9 Pancreatic cancer

Pancreatic cancer (PC) affects the pancreas, a glandular organ in the abdomen behind the stomach, which plays an essential role in digestion and regulating blood sugar levels. Pancreatic cancer occurs when cells in the pancreas start to grow and divide uncontrollably, forming a tumor. Pancreatic cancer can be challenging to diagnose in its early stages, as symptoms may not appear until it spreads to other body parts. Treatment for pancreatic cancer may include surgery, radiation therapy, chemotherapy, or a combination of these therapies, depending on the stage and location of cancer. The prognosis for pancreatic cancer can be poor, mainly if the cancer has spread to other body parts.

TRAP1 can upgrade the tumor cell death threshold and give them resistance to antineoplastic therapy by scavenging harmful ROS (89, 90, 181, 182) and suppressing mitochondrial permeability transition pore (mPTP) cyclophilin D (CypD; PPIF), which is en essential survival mechanism (14).

Hsp90 inhibitors may enhance pancreatic cancer cells’ cytotoxic sensitivity by causing client protein degradation (183). Lang et al. reported that blocking Hsp90 can cause growth-inhibitory by obstructing insulin-like growth factor-I (IGF-I) and interleukin-6 (IL-6), proangiogenic signaling cascades (184). Besides, IPI-504, an Hsp90 inhibitor, showed an anti-proliferative effect on PC growth (185). Moreover, Xianhua Cao introduced a combination therapy of geldanamycin and 3BrPA that enhances efficacy and reduces dose-limitation toxicity to treat chemotherapy-resistant PC (186, 187). Tarik Ghadban et al. demonstrated that Hsp90 inhibitors (17-AAG and 17-DMAG) could disrupt gemcitabine and 5- fluorouracil signal cascades in PC, which can promote tumor cell apoptosis (188). These studies showed promising prospects, but few clinical trials assessed them. To date, only one phrase II trial reported that it is not warranted to study targeting Hsp90 inhibiting drugs with gemcitabine in PC treatment (183).

Though there is not enough targeting TRAP1 inhibitors for PC treatment, the ATPase in NTD of other Hsp90 inhibitors have similar structure with TRAP1, one has proved that these Hsp90 inhibitors can competitively inactive TRAP1 ATPase *in vitro* (189), which can be used to develop TRAP1 selective inhibitors in future.

## 5 Discussion

Despite the increasing maturity of our understanding of mtHsp90, there are still unresolved concerns that require further investigation. Firstly, it is imperative to evaluate the potential relevance of ATPase activity to the function of TRAP1. That’s because previous studies have demonstrated that TRAP1, even in a catalytically inactive state, is capable of carrying out its role and reversing mitochondrial dysfunction (26). Secondly, as for how mtHsp90 regulate tumorigenesis and therapy resistance of various cancers at the molecular level, our cognition is still limited. In the future, we may focus more on the molecular functional mechanisms and related signally pathways with mtHsp90. Furthermore, given the lack of clinical success observed in numerous cytosolic Hsp90 inhibitors, it may be prudent to explore the possibility of devising a strategy to concurrently disrupt the mtHsp90 pool in order to evaluate their combined efficacy. MtHsp90 inhibitors have demonstrated the ability to effectively control cancer cell growth both *in vitro* and *in vivo*. There are several mtHsp90 inhibitors, such as gamitrinib, SMTIN-P01, SMTIN-C10, and DN401, that have been successfully designed to target mitochondria (**Table 1**) (5, 31, 119, 191). We have reviewed the research progress of mtHSP90 inhibitors for the treatment of relevant cancers according to the cancer types, which will be helpful for the development of therapeutic drugs for different cancers. Meanwhile, the synthesis and exploration of new Hsp90 drugs are also ongoing, as explained in the reviews by Dernovšek et al. (19) and Xie et al. (85). Moreover, mtHsp90 inhibitors have the potential to selectively accumulate in the mitochondria of cancer cells and limit toxicity to normal tissues. This effectively addresses the primary challenge of other drugs that target the entire Hsp90 in mammals. Another promising area of research is the combination of mtHsp90 inhibitors with other cancer therapies. Recent studies have demonstrated that mtHsp90 inhibitors can enhance the efficacy of other drugs, such as chemotherapy, radiation therapy, and immunotherapy. Combining these therapies could result in more effective treatment regimens with lower toxicity and better patient outcomes. However, despite the preclinical trials continually demonstrating the feasibility and universal validity of mtHsp90 inhibitors, there is still a lack of clinical trials in humans (**Table 2**). Further research is needed to ensure the feasibility of these inhibitors before human testing.

**Table 1:** Model systems in which mtHsp90 inhibitors were applied successfully, resulting in cell death or growth inhibition.

Cancer type	Cancer cell line *in vitro*	Animal models	Inhibitors tested	Ref.
Prostate cancer	DU145		gamitrinib	(119)
	22Rv1 and PC3	22Rv1 xenograf	gamitrinib	(90)
	PC3	PC3 Superficial prostate cancer xenograft model, PC3 Orthotopic bone metastatic prostate cancer model	gamitrinib	(126)
	RM1	TRAMP mice	gamitrinib	(127)
	PC3		gamitrinib	(190)
	PC3, LNCaP	TRAMP mice	gamitrinib	(6)
	22Rv1 and PC3		SMTIN-P01	(5)
	PC3		DN401	(191)
	PC3	PC3 xenograft Model	SMTIN-C10	(31)
	DU-145		CVM-1118	(122)
	PC3	PC3 xenograft model	pyrazolo[3,4-d] pyrimidine derivatives	(52)
	PC3		6BrCaQ-Cn-TPP conjugates	(180)
Lung cancer	H460, H1975	H460 lung xenograft mouse model	gamitrinib	(119)
	H1299, H2122, H358, H2073, H460, H2347, H1975, H1395 and H2085		gamitrinib	(108)
	NCI-H460		gamitrinib	(90)
	H1299, A549, H1437, and H1650		gamitrinib	(6)
	NCI-H460		SMTIN-P01	(5)
	NCI-H460		SMTIN-C10	(31)
	A549		CVM-1118	(122)
	NCI-H460		pyrazolo[3,4-d] pyrimidine derivatives	(52)
	NCI-H460		IR-PU	(120)
Breast cancer	MDA-MB-231, MCF-7	MDA-MB-231 breast cancer xenograft mouse model	gamitrinib	(119)
	MDA-MB-231	MDA-MB-231 xenograf mouse	gamitrinib	(90)
	MCF-7		gamitrinib	(6)
	MDA-MB-231		SMTIN-P01	(5)
	MDA-MB-231		DN401	(191)
	BC MCF7		gamitrinib	(160)
	MDA-MB-231, MDA- MB-435, MCF-7		CVM-1118	(122)
	MDA-MB-231		pyrazolo[3,4-d] pyrimidine derivatives	(52)
	MDA-MB-231		6BrCaQ-Cn-TPP conjugates	(180)
Glioblastoma	U87MG		gamitrinib	(119)
	LN229		gamitrinib	(190)
	LN229		gamitrinib	(6)
	U251MG, A172, M059K, H4, Hs683, M059J, LN18, LN229, U87MG, U118MG, U138MG, DBTRG-05MG, and T98G; D2363PXA, D645PXA, D2159MG, and D2224MG		gamitrinib	(135)
	U87MG, LN229, U251 and T98G	xenograft mouse model	gamitrinib-tpp	(138)
	U87, LN229, T98G, U251, GBM12, GBM14 and GBM43	GBM12 and GBM43 xenograft mouse model	gamitrinib	(137)
	LN229、U87、T98G、GBM12、GBM22		gamitrinib	(136)
	SHG44, U251−MG and U87−MG		gamitrinib-tpp	(134)
	U118MG		CVM-1118	(122)
Colorectal cancer	HCT116		gamitrinib	(136)
	DLD1, RKO, SW48, HT29 and HCT116		gamitrinib	(162)
	RC HCT116, HT29, COLO320, COLO205 and CaCo2		gamitrinib	(160)
	HT-29, HCT-116, COLO205		CVM-1118	(122)
	HCT116		Gamitrinib-TPP	(161)
	HT-29, HCT-116		6BrCaQ-Cn-TPP conjugates	(180)
Leukemia		HL60 leukemia xenograft mouse model	gamitrinib-tpp	(191)
	K562, Raji, THP-1, HL-60	HL60 leukemia xenograft mouse model	gamitrinib-g4	(125)
	K562		6BrCaQ-Cn-TPP conjugates	(180)
Liver cancer	SK-HEP-1		pyrazolo[3,4-d] pyrimidine derivatives	(52)
Gastric cancer	AGS		vacA	(147)

**Table 2:** Clinical trials for mtHSP90 inhibitors.

Drug	Date	Phase	Clinical trials.gov
gamitrinib-TTP	Estimated Primary Completion Date: December 2028	PhaseI	NCT04827810
CVM-1118	Actual Study Completion Date: April 8, 2022	PhaseI	NCT04336124
	Estimated Primary Completion Date: September 2025	PhaseII	NCT05257590

We anticipate further testing of mtHsp90 inhibitors in a broader range of cancer types beyond those mentioned in this review. While the efficacy of inhibitors targeting the other Hsp90 protein, such as Ganetespib (STA-9090) and luminespib (AUY922), has been demonstrated in certain cancers, including hepatocellular carcinoma (HCC) (192), pancreatic cancer (PC) (193), gastric cancer (GC) (194, 195).

However, there is a lack of research on the use of mtHsp90 inhibitors in hepatocellular carcinoma, pancreatic cancer, gastric cancer (188, 196, 197). Thus, further testing focused on mtHsp90 inhibitors may prove valuable in identifying new strategies for controlling these cancers. In addition, some studies found that the combination of mtHsp90 inhibitors with other available drugs may represent a promising approach to enhance the anticancer activity of different therapies, while minimizing their undesirable side effects. Hence, the combination of mtHsp90 inhibitors with other drugs may unlock their full potential as anticancer agents and expand their use to a wider range of cancer types.

In summary, the prospective therapeutic application of mtHsp90 inhibitors in the treatment of cancers is highly promising. Nevertheless, further investigation is needed to comprehensively ascertain their effectiveness and safety in further studies.

## 6 Conclusion

This review presents a concise summary of the involvement of mtHsp90 in the progression of cancer. The investigation and development of mtHsp90 inhibitors have exhibited their capacity to significantly revolutionize the field of cancer therapy and enhance the overall prognosis of patients. Researchers have demonstrated promising results in utilizing gamitrinib and other mtHsp90 inhibitors as a novel approach to combat cancer, both *in vitro* animal models including prostate cancer and breast cancer, as well as *in vivo* cancer cell lines. Nevertheless, it is important to recognize that there exist numerous obstacles that require resolution. Further investigation in this field has the potential for making breakthroughs in the realm of cancer treatment, leading to the creation of more secure and effective therapeutic approaches in years to come.

## Author contributions

YX: Writing – original draft, Writing – review & editing. XuL: Writing – original draft, Writing – review & editing. QS: Writing – original draft, Writing – review & editing. KL: Writing – original draft. XiL: Writing – original draft. ZZ: Writing – original draft. LF: Writing – original draft. YL: Writing – review & editing. BW: Writing – review & editing.
